# Social polymorphism in the sweat bee *Lasioglossum* (*Evylaeus*) *calceatum*

**DOI:** 10.1007/s00040-016-0473-3

**Published:** 2016-05-10

**Authors:** P. J. Davison, J. Field

**Affiliations:** School of Life Sciences, University of Sussex, John Maynard Smith Building, Brighton, BN1 9QG UK; Carl Icahn Laboratory, Lewis-Sigler Institute for Integrative Genomics, Princeton University, Washington Road, Princeton, NJ 08544 USA

**Keywords:** Sweat bees, Halictinae, *Lasioglossum*, Eusociality, Caste-size dimorphism, Workers

## Abstract

Temperate-zone socially polymorphic sweat bees (Hymenoptera: Halictidae) are ideal model systems for elucidating the origins of eusociality, a major evolutionary transition. Bees express either social or solitary behaviour in different parts of their range, and social phenotype typically correlates with season length. Despite their obvious utility, however, socially polymorphic sweat bees have received relatively little attention with respect to understanding the origins of eusociality. *Lasioglossum* (*Evylaeus*) *calceatum* is a widespread sweat bee that is thought to be socially polymorphic, with important potential as an experimental model species. We first determined the social phenotype of *L. calceatum* at three sites located at different latitudes within the UK. We then investigated sociality in detail across two years at the southernmost site. We found that *L. calceatum* exhibits latitudinal social polymorphism within the UK; bees were solitary at our two northern sites but the majority of nests were social at our southern site. Sociality in the south was characterised by a relatively small mean of two and 3.5 workers per nest in each year, respectively, and a small to medium mean caste-size dimorphism of 6.6 %. Foundresses were smaller in our more northern and high altitude populations. Sociality is clearly less specialised than in some closely related obligately social species but probably more specialied than other polymorphic sweat bees. Our research provides a starting point for future experimental work to investigate mechanisms underlying social polymorphism in *L*. *calceatum*.

**Electronic supplementary material:**

The online version of this article (doi:10.1007/s00040-016-0473-3) contains supplementary material, which is available to authorized users.

## Introduction

Understanding why an individual gives up its own reproduction to help others is a central goal of evolutionary biology (Maynard Smith and Szathmáry 1995). Despite this, accounting for how the decision to help is made at the individual level has received comparatively little attention (Field et al. 2012). Primitively eusocial sweat bees (Hymenoptera: Halictidae) lack fixed castes and vary greatly in their social organisation, making them model organisms for studying the origins of eusociality (Schwarz et al. 2007). Of particular interest is social polymorphism, where both solitary and social phenotypes are expressed within the same species (Soucy and Danforth 2002). In social nests, at least some first brood offspring are workers that help rear a second brood of reproductives. In contrast all offspring in solitary nests are reproductives, which tend to occur where the season is probably too short to complete the social life cycle (Hirata and Higashi 2008; Kocher et al. 2014). Socially polymorphic sweat bee lineages therefore offer unique opportunities to understand the mechanisms underlying the origin of eusociality, because it is possible to directly investigate the environmental and genetic processes mediating the decision to become a worker or a reproductive (Field et al. 2010, 2012).

The Palearctic sweat bee *Lasioglossum* (*Evylaeus*) *calceatum* Scopoli is socially polymorphic (Sakagami and Munakata 1972; Field 1996). Originally this species was thought to be only primitively eusocial (e.g. Bonelli 1965, 1968). Then, more than 40 years ago, Sakagami and Munakata (1972) discovered that *L. calceatum* was socially polymorphic in Japan; nests were found to be solitary at more than 1000 masl on the summit of Mt Yokotsu, but a social life cycle was inferred in the surrounding lowlands. Similarly, Field (1996) reported solitary nests from Dartmoor, an upland area in the southern United Kingdom (UK). Since these studies, however, there has been no attempt to further understand the underlying causes of social polymorphism within *L. calceatum*. Moreover, details of the social life cycle and the degree of social complexity relative to other primitively eusocial sweat bees remain poorly understood from the wild (Plateaux-Quénu 1992; Pesenko et al. 2000).

From studies to date, the life cycle of *L. calceatum* can be summarised as follows (Bonelli 1965, 1968; Sakagami and Munakata 1972; Plateaux-Quénu 1992 and references therein). Mated females (foundresses) emerge from hibernation in spring and initiate a subterranean nest. Foundresses mass provision a first brood (B1) of ≈4–6 offspring including both females and males, providing each with a ball of pollen and nectar in a cluster of separate, sealed brood cells. In solitary nests offspring emerge, mate, and females enter directly into hibernation. In social nests, however, B1 females are typically slightly smaller than their mothers and are thought to become workers that help provision a second brood (B2) of reproductives. This conclusion is supported by field data from Europe and Japan; summer caught females are reported to be mostly unmated and to have undeveloped ovaries (Bonelli 1965; Sakagami and Munakata 1972, but see Plateaux-Quénu 1992 who reports a greater proportion of mated summer females). B2 offspring emerge at the end of summer to mate, and females enter hibernation before emerging as foundresses the following spring. Males are produced in both broods but die before winter and play no role in nesting. Nevertheless, B1 sweat bee offspring may also assume replacement queen status, lay eggs in nearby nests, found a nest directly or enter hibernation to become a foundress in the following year (Yanega 1988; Yagi and Hasegawa 2012; Brand and Chapuisat 2016). It is currently unknown to what extent these behaviours occur in *L. calceatum*.

More advanced halictine sociality is generally associated with traits such as larger colony size, and a greater degree of caste-size dimorphism between workers and foundresses (Packer and Knerer 1985). A population level comparison between foundresses and presumed B1 females in Japanese *L. calceatum* found a size difference of 3.5–5.5 % (Sakagami and Munakata 1972), whereas in France foundresses have been reported as being up to 13 % larger than workers (see Plateaux-Quénu 1992). It is unclear whether the latter figure was also measured at the population level or directly between mothers and daughters within nests, but these data do indicate there may be geographic variation in caste-size dimorphism. Colony size in wild nests has been reported from only a single location in Italy, where Bonelli (1965) excavated nests with 4-6 B1 brood cells.

Characteristics such as caste-size dimorphism, number of workers and bee size may vary temporally as well as spatially, and can be influenced by fluctuating environmental conditions. In a multiyear study of *Halictus ligatus* , say, such characteristics largely depended upon weather conditions from year to year (Richards and Packer, 1996). For example, the sizes of a foundress and her workers are determined in separate years. A large foundress may be produced in a dry, warm year but then raise small workers if the subsequent year is cool and wet (Richards and Packer 1996). Consequently it is not only necessary to study geographically disparate populations, but also individual populations over multiple years to achieve an accurate description of social phenotype (Wcislo 1997).

The geographic distribution of social and solitary phenotypes within polymorphic species is closely associated with the length of the active season. Bees typically nest socially in southern and low altitude areas where the season is long enough to facilitate rearing two broods (Soucy 2002; Field et al. 2010), but solitary at higher latitudes or altitudes where multiple broods are likely to be temporally precluded (Eickwort et al. 1996; Field 1996). Recent work on *Halictus rubicundus* Christ in the United Kingdom (UK) has shown that social phenotype was plastic, and that time of first brood emergence could be an important factor influencing whether offspring become workers (Field et al. 2010). However, because foundresses are capable of varying the size of B1 offspring with respect to expected social phenotype (Field et al. 2012, but see Field et al. 2010), they may use a reliable cue such as time of nest initiation to inform whether or not they provision smaller, worker-sized offspring. The timing of nest initiation could therefore be an important factor determining social phenotype if earlier-provisioned offspring are themselves likely to emerge earlier, and foundresses could therefore use time of provisioning to anticipate social phenotype (Field et al. 2010). Nevertheless, the generality of this pattern is not clear (e.g. Yanega 1993; Field et al. 2012).

**Fig. 1:**
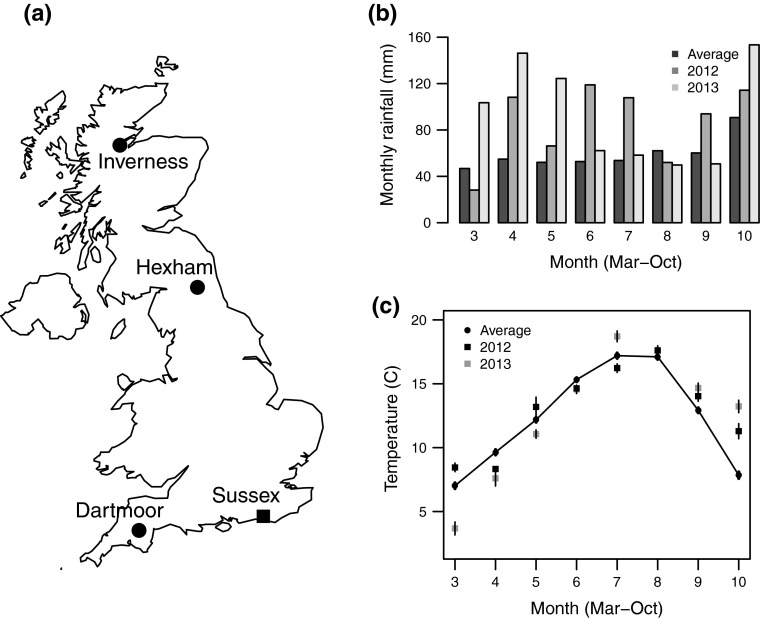
**a** *Map* showing the locations of the University of Sussex campus (Sussex), Hexham, Dartmoor and Inverness. *Circles* denote sites where bees are solitary, and the *square* where bees show primitive eusociality. **b** Average monthly rainfall for southeast England between March and October (1990–2015), and total monthly rainfall at Sussex in 2012 and 2013. Dark *grey* *bars* show the long-term average, medium grey are 2012 and *light* *grey* *bars* are 2013. **c** Average monthly temperature for southeast England between March and October (1990–2015), and mean daily temperature per month at Sussex in 2012 and 2013. *Dark* *circles* connected with the line show the long-term average, *dark* *squares* are 2012 and *light* *squares* are 2013. Temperatures are presented ±1SE

In the present study we determine whether *L. calceatum* is socially polymorphic in the UK, by establishing social phenotype at three different latitudes (Table 1; Fig. 1a). We also investigate in detail the social phenotype of *L. calceatum* over 2 years at the southernmost site (Sussex). At Sussex we investigate bee size, caste-size dimorphism, and the number of workers, and test for a relationship between the date on which a foundress begins provisioning in spring and the date on which her first female offspring emerges. We also compare bee size among sites, using additional specimens from a population on Dartmoor that is thought to be solitary (Fig. 1a; Field 1996).

**Table 1:** Details of the sites used in the study

Location	Latitude/longitude	Temperature °C^a^	Altitude (masl)	Year studied	Number of nests at start of spring	Number of observation days
Sussex	50.864 N/-0.084 W	17.4	82	2012–2013	>100 (2012) <40 (2013)	120 (2012) 50 (2013)
Hexham	54.978 N/-2.100 W	14.4	37	2012	≈20	NA
Inverness	57.554 N/-4.456 W	13.4	5	2013–2014	>100 both years	NA
Dartmoor	50.5 N/-3.8 W	16	>300	1992^b^	NA	NA

Temperature data are the mean annual land surface temperatures for each site

^a^Mean land surface temperature 1981–2006 (Hay et al. 2006)

^b^See Field (1996)

## Methods

### Study sites

Three nesting aggregations of *L. calceatum* in the UK where social phenotype was previously unknown were studied between 2012 and 2014 (Table 1; Fig. 1a). Details of each site are given in Table 1. The Sussex site was a narrow, west-facing strip of grass 5.8 m long and 1.3 m wide on the University of Sussex campus, bordered on the eastern side by a single storey brick building. The site at Hexham was a small section of a much larger south-facing recreational grassland area approximately 5 m long and 3 m wide, bordered on the northern side by a row of mature trees. At Inverness, nests were situated in the grassy centre and to the sides of a 5 m section of stone track. Sweat bee nesting aggregations are notoriously difficult to find (Richards et al. 2015). Therefore, to preserve our study sites for future experimental work we did not destructively sample any bees or excavate nests, and the present study focuses on behavioural observations.

### Method of observation at Sussex

**Fig. 2:**
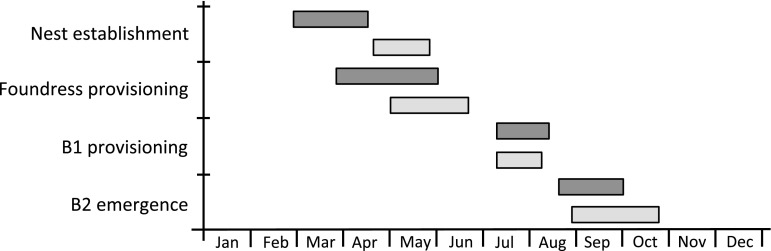
Nesting cycle of *Lasioglossum calceatum* on the University of Sussex campus in 2012 (*dark* *grey* *bars*) and 2013 (*light* *grey* *bars*). Temporal overlap between stages within each year represents periods when not all bees were at the same stage

Detailed observations were made to establish and characterise the social phenotype of *L. calceatum* at Sussex. Behaviour was observed directly by continuously standing or sitting in front of the aggregation for the duration of activity on every day of suitable weather (Observation days; 2012, *n* = 120, 2013 *n* = 50). In the early spring of 2012 and 2013 the aggregation was checked daily on sunny days for activity by newly emerged foundresses. The first *L. calceatum* foundress was seen on 29 February and 20 April in 2012 and 2013, respectively, and activity continued until October in both years (Fig. 2). Continuous observations in 2012 and 2013 commenced from these dates in each year, respectively, thereby ensuring that we observed the first provisioning date for each foundress.

In 2012 a subset of 50 foundresses from 47 nests was marked and measured during the foundress-provisioning phase, and in 2013, 23 foundresses from 17 nests within the observation area were marked and measured. Foundresses were caught with an insect net as they emerged from their burrows after a provisioning event had been observed. Each was given a unique combination of enamel paint spots (Revell^®^ and Humbrol™ enamel model paints) applied to the thorax with a pin. Wing length was measured to the nearest 0.1 mm with digital callipers, as the distance between the outer edge of the tegula and the end of the forewing. The aggregation was divided into two sections, and these were observed on alternate days.

Sociality was confirmed by the presence of workers. These were identified as unmarked bees observed provisioning the nests (where the foundress or foundresses had been marked) after the short period of inactivity between foundress-provisioning and offspring emergence (Fig. 2). Workers were caught and measured on departure from their nest after a provisioning event had been recorded. Within each nest, workers were given a single unique colour spot. A bee was designated as a worker only if it was observed provisioning again after marking; the total number of workers for each nest was counted as all such bees. Provisioning events within each area were recorded, and where possible the colour of the provisioning worker noted.

### Methods of observation at Hexham and Inverness, and Dartmoor foundresses

Aggregations at Hexham and Inverness were first visited during the foundress-provisioning phase on 19–20 June 2012 and 3–4 June 2013, respectively. Foundresses were caught in flight at Hexham (*n* = 17), and marked with a single colour spot until no unmarked provisioning bees remained. At Inverness ten foundresses were marked and their nest locations noted. Wing length for all marked specimens was measured. A second visit to Hexham was made on 27 June 2012, and repeat visits to Inverness on 10–11 July 2013, 20–21 August 2013, and 10–11 September 2013 to check for the presence of workers. Foundresses were not marked at Inverness in 2014, but visits were made throughout August to check for provisioning workers. In addition, wing lengths of specimens from a population on Dartmoor caught in 1992 by J. Field were also measured. Dartmoor is an area of upland generally >300 masl in the southwest UK, where *L. calceatum* is thought to nest solitarily (Field 1996; Table 1; Fig. 1a).

### Climate and weather data

Weather data at each site were taken from nearby web-based weather stations (each no more than ±30 m difference in altitude from the respective study site) located on Weather Underground (http://www.wunderground.com). For Sussex the nearest station was in Lewes, 5.8 km away (station IDNS52). For Hexham the nearest station was in Hexham (station INORTHUM28), approximately 2 km from the site, and for Inverness the nearest station was less than 1 km away (Station IROSS-SH1) in Maryburgh. Weather stations were approximately the same altitude as the field site). Rainfall and temperature data for the southeast of England were downloaded from the UK Meteorological Office website (http://www.metoffice.gov.uk) to construct long-term averages (1990–2015). Temperature data presented in Table 1 are the 1981–2001 annual mean land surface temperature derived from the satellite-mounted Advanced Very High Resolution Radiometer (AVHRR) sensor (Hay et al. 2006).

### Data analysis

Unless stated otherwise all analyses incorporate data from both 2012 and 2013. Supporting data are available in the online supplementary materials. Interaction terms between other explanatory variables and year were initially included in maximal models. These were never significant and are not reported. We generally report the main effect of ‘year’ as a covariate where significant only. All analyses were conducted in the R environment (R Development Core Team 2013), using the lme4 package (Bates et al. 2015) for generalised linear mixed models (GLMMs). Results are presented ±1 standard error.

Environmental variation is known to affect the nesting success of ground-nesting Hymenoptera, with excessive rainfall causing elevated brood mortality (Richards and Packer 1995; Soucy 2002). Patterns of rainfall were different between years (Fig. 1b), and we used this opportunity to examine the effect of weather on nesting success and B1 productivity. Nest co-founding has been shown to reduce the chances of nest failure (Richards and Packer 1998), here defined as failure to produce any detected B1 offspring. We use a generalised linear model (GLM) with binomial and normal errors to investigate whether nest failure rates and the number of workers produced differed between years, and whether co-founded nests were less likely to fail. Further, we also use a GLM with binomial errors to investigate whether foundress size affected nest failure. For this analysis, co-founded nests were excluded because it was not known whether the mother to the offspring had been measured.

Foundresses might use date of first provision in spring as a cue for offspring emergence time, if earlier-provisioned offspring emerge earlier in the year (Field et al. 2012). We use a generalised linear model (GLM) with normal errors to test for a relationship between a foundress’ first provisioning date and the date of her first B1 offspring emergence. Earlier-starting foundresses may produce more workers because they have more time during which to provision, and/or larger foundresses may produce more workers because they are better at foraging. We therefore use a GLM with Poisson errors to test the effect of foundress size and date of first provision on the number of workers produced. Co-founding may also increase B1 productivity, and we therefore also use a GLM with Poisson errors to examine whether co-founded nests produced more workers than singly founded nests.

We used a GLMM with normal errors to test for significant differences in wing length between foundresses and workers, with ‘caste’ and ‘year’ as fixed factors and ‘nest’ as a random factor. We include only those foundresses that produced workers. Within-nest caste-size dimorphism was calculated after Packer and Knerer (1985) as [((*F*−*W*)/*F*) × 100)], where *F* is foundress wing length and *W* is worker wing length.

We use a one-way ANOVA to test for differences in foundress wing length between sites, and Tukey’s HSD test to determine significant differences between sites. Foundresses from ‘Inverness’ include additional samples from other nearby aggregations that were not studied (*n* = 6 from the ‘Inverness’ study site, *n* = 5 from other sites less than 10 km away). All foundresses from both years at Sussex were included regardless of whether they produced offspring. Analyses of foundress and worker size, foundress size and the number of workers, and caste-size dimorphism excluded co-founded nests, because it was not known which bee was mother to the offspring. In 2013 three bees that had previously been co-foundresses later initiated their own nests independently, and began provisioning during a week when observations were not being made. These three nests were therefore assigned a provisioning start date of the first day of that week.

Finally, to place *L. calceatum* sociality in a broader context it is useful to compare our results with published data from other closely related species. With additional data from more recently published work and the present study we follow Bourke (1999) and use Spearman’s rank correlation coefficient to test for a relationship between worker brood size and caste-size dimorphism within the *Lasioglossum* subgenus *Evylaeus* (see Table S1 in supplementary material data and sources). To ensure measures of caste-size dimorphism were comparable, we conducted separate analyses on data from studies where body size had been measured as wing length or head width, respectively. The number of workers in all but one of the other studies listed in Table S1 is based on nest excavations rather than observations, as in the present study.

## Results

### Nest-founding and nesting success

Foundresses were recorded provisioning at 100 nests in 2012, and 27 nests in 2013. March was considerably warmer in 2012 than in 2013, leading to an extended period of foundress emergence in 2012. In contrast, the spring of 2013 started later, resulting in a shorter foundress emergence period (Figs. 1c, 2). After the early start in 2012, the weather deteriorated and was very wet for much of the remaining spring and summer (Fig. 1b). After foundress emergence in 2013, however, the weather was much drier with extended periods of sunshine and a summer heat wave (Fig. 1b, c). Reflecting this, a significantly greater proportion of nests failed to produce any detected B1 offspring in 2012 than in 2013 (GLM: $X^{ 2}_{ 1, 1 2 5}$ = 19.578, *p* < 0.001; 2012: 84 %, *n* = 84 failed, *n* = 16 successful, 2013: 37 %, *n* = 10 failed, *n* = 17 successful). A small proportion of nests were co-founded (5 % (5/100) in 2012, 16 % (4/27) in 2013) with up to three provisioning co-foundresses in a nest. Co-founded nests were significantly more likely to produce detected B1 offspring than singly founded nests (GLM: $X^{ 2}_{{ 1,{ 125}}}$ = 4.719, *p* = 0.030; 2012 *n* = 3/5 (60 %) succeeded, 2013: *n* = 2/4 (50 %) succeeded).

### Social phenotypes detected

At Sussex, social phenotype was determined at 16 nests in 2012 and 17 nests in 2013. The life cycle is summarised in Fig. 2. B1 offspring in most nests appeared to be social: unmarked bees and began provisioning upon emergence subsequent to the activity break after foundress-provisioning. At two nests in 2012 (12.5 %), offspring appearing to be solitary: unmarked females repeatedly entered and left the nests on multiple days, but never provisioned. They then disappeared, presumably to enter hibernation, which we have since directly observed (Davison and Field in prep.). These nests were therefore probably solitary. In both cases the original nest foundress was still alive, but neither resumed provisioning. All offspring at two further nests in 2012 were also possibly solitary, although it was less clear because the foundresses were unmarked. All nests (*n* = 17) in 2013 with detected B1 offspring were social, although we could not detect whether any B1 females additionally entered hibernation. The conclusion that B1 offspring were predominantly behaving as workers is supported by data from a subsequent year at Sussex, in which microsatellite genotypes suggests that a single egg-layer typically monopolised B2 reproduction within each nest (Davison and Field in prep.).

One individual marked as a worker in 2012 reappeared during the nest-founding phase of 2013, confirming that some B1 offspring entered directly into hibernation. Most offspring entering directly into hibernation would not have been marked if they did not start provisioning. In both years at Sussex unmarked bees were observed leaving and entering nests after all workers there had been marked. Additionally, unmarked bees were observed flying around the aggregation as foundresses do in spring when searching for a nesting site: it is possible these were early hibernating B1 females, although intraspecific parasitism cannot be discounted.

Bees at both Hexham and Inverness were solitary. At both sites B1 offspring were observed returning to many different nests, but provisioning was not observed at any nest. The sampling method employed at Hexham and Inverness is unlikely to have overlooked social nests, as there were no days at Sussex on which multiple nests were active where B1 provisioning was not observed. Some nests at Inverness and Sussex were co-founded but observations to test for this were not made at Hexham.

### Foundress-provisioning and B1 emergence

**Fig. 3:**
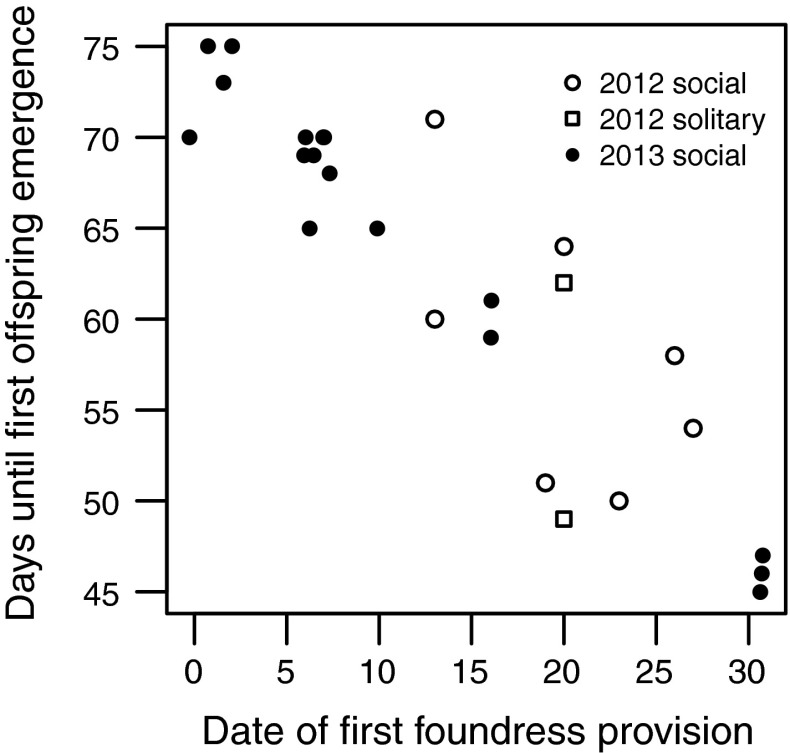
Relationship between the date on which a foundress first provisioned and the number of days until her first B1 offspring emerged. Data are shown for both years. Open symbols are data from 2012: *circles* show nests where offspring became social, and *squares* show nests that were probably solitary. *Filled* *circles* are data from 2013. The later a foundress began provisioning, the shorter the time before her first offspring emerged. Day 0 is April 30. Individual points are horizontally jittered to show overlapping data

The time between the date of a foundress’ first recorded provisioning trip and the date of her first B1 offspring emergence decreased linearly with date of first provision (GLM: F_1,25_ = 115.49, *p* = 0.001; Fig. 3). This pattern meant that earlier-provisioning foundresses did not produce offspring that emerged earlier (GLM: F_1,25_ = 2.704, *p* = 0.113). Foundresses that began provisioning earlier did not produce more workers (GLM: F_1,25_ = 2.704, *p* = 0.335). Foundresses were never observed provisioning after the emergence of their B1 offspring.

### Bee size and number of workers

**Fig. 4:**
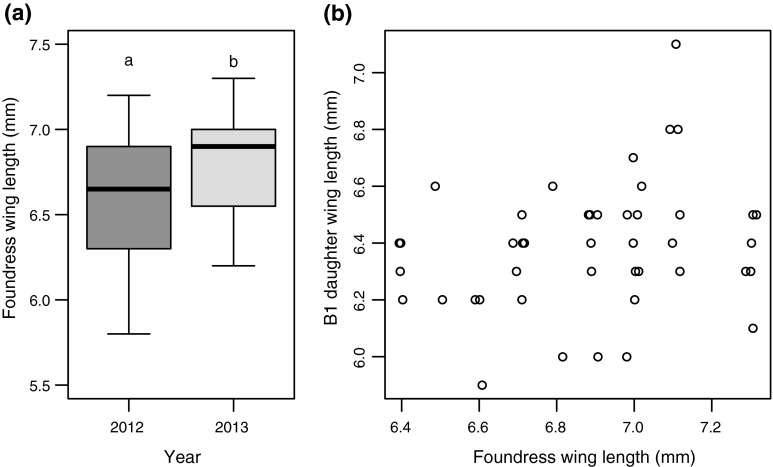
**a** The wing length of all measured foundresses in the spring of 2012 and 2013. *Letters* above boxes denote significant differences (see text). **b** Relationship between the wing lengths of foundresses and the B1 daughters they produced. Individual points are horizontally jittered to show overlapping data

Foundresses that emerged and initiated nests in the spring of 2012 were significantly smaller than foundresses in 2013 (Fig. 4a; *t* test: *t* = −2.389, *p* = 0.021, 2012 *n* = 51, 2013 *n* = 23). Foundresses produced workers significantly smaller than themselves (*n* = 18 nests, GLMM: $X^{ 2}_{ 1}$ = 51.655, *p* < 0.001), with a mean within nest caste-size dimorphism of 6.6 % based on wing length (foundresses = 6.88 mm ±0.06, workers = 6.39 ±0.03). However, of foundresses that produced offspring there was no effect of year (GLMM: $X^{ 2}_{ 1}$ = 0.011, *p* = 0.918) such that successful foundresses and the workers they produced did not differ in size between years. Despite the fact that foundresses overall were larger in 2013 (Fig. 4a), we could not detect any effect of foundress size on nesting success (GLM: $X^{ 2}_{ 1, 5 6}$, *p* = 0.253). Among nests, caste-size dimorphism ranged from 0 to 13 %, with four nests containing one or more workers that were the same size as the foundress, and there was large size overlap between castes. Larger foundresses did not produce larger workers (Fig. 4b; GLMM: $X^{ 2}_{ 1}$ = 2.443, *p* = 0.295), and due to this pattern caste-size dimorphism within nests tended to vary relative to the size of the foundress. Larger foundresses also did not produce more workers (GLM: $X^{ 2}_{ 1}$ = 0.451, *p* = 0.502).

Foundresses produced a mean of 2 ± 0.36 workers in 2012 (range 1–5) but were significantly more productive in 2013, producing 3.5 ± 0.42 in 2013 (range 1–4) (*n* = 17 nests in both years, Wilcoxon rank-sum test: *W* = 70, *p* = 0.009). Co-founded nests, however, did produce significantly more workers than singly founded nests (GLM: $X^{ 2}_{ 1}$ = 6.940, *p* = 0.004, singly founded = 2.35 ± 0.30, co-founded = 4 ± 1.09).

### Natural enemies

The halictid cuckoo parasite *Sphecodes* was continuously present in small numbers at Sussex in 2012, and a single parasitic fly (species unknown) was observed following a foundress to her nest and subsequently entering. Bee flies (*Bombylius*), known to parasitize *Lasioglossum* (Wyman and Richards 2003; Boesi 2009), were also present at the aggregation during spring. Only a single *Sphecodes* female was observed in the spring of 2013. This was caught and later identified as *S. monilicornis*, known to be a cuckoo of *L. calceatum* (Bogusch et al. 2006 and references therein). Ants (*Lasius* sp.) attacked nests during the foundress-provisioning phase, preventing foundresses from entering their nests with pollen, and also raided nests during the B1 worker phase.

### Geographic size variation

**Fig. 5:**
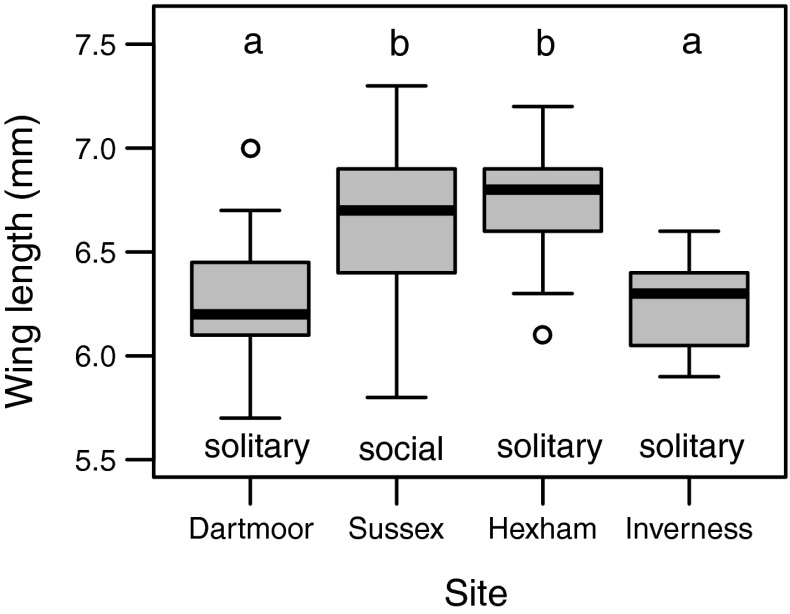
Geographic variation in the wing length of foundresses from sites with different social phenotype. The predominant social phenotype at each site is given below each box. *Letters* above the boxes denote significant differences (see text)

There was a significant effect of site on foundress size (one-way ANOVA: F_4,111_ = 9.372, *p* < 0.001). Tukey’s HSD test revealed that foundresses from both Dartmoor and Inverness were significantly smaller than those from Sussex and Hexham (Fig. 5; see Table S1 for pairwise comparisons).

### Social level in *Evylaeus*

**Fig. 6:**
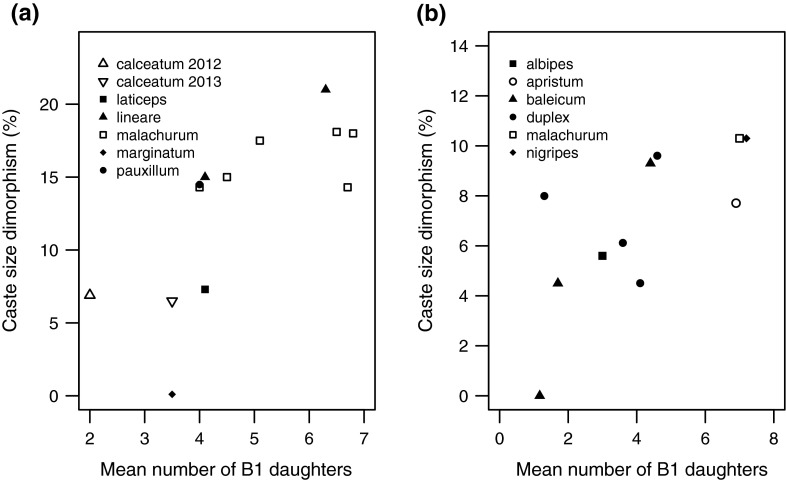
Significant positive correlation between the mean number of B1 daughters produced and caste-size dimorphism among species in the *Lasioglossum* subgenus *Evylaeus*, where body size was measured as (**a**) wing length or (**b**) head width. Note the different y-axis scales. Species names are given in each legend (see table S1 for data sources)

There was a significant positive correlation between the number of B1 workers and caste-size dimorphism, whether size was measured as wing length (Fig. 6a; Spearman’s rank correlation: *r* = 0.775, *n* = 13, *p* = 0.002) or head width (Fig. 6b; Spearman’s rank correlation*: r* = 0.776, *n* = 11, *p* = 0.005). The less socially specialised species tend towards the lower left of Fig. 6a, b, and the more specialised the upper right. Results from Sussex place *L. calceatum* in the lower left portion of Fig. 6a, indicating that it is relatively less socially specialised than other members of the subgenus.

## Discussion

Socially polymorphic sweat bees are of particular interest for elucidating the behavioural and genetic changes associated with the origins of eusociality (Chapuisat 2010). Nevertheless, few studies have focussed on temperate-zone socially polymorphic species. In the present study, we determined the social phenotype of *L*. *calceatum* at different latitudes in the UK, and investigated its life cycle across 2 years at Sussex, the southernmost site (Table 1; Fig. 1a). Our results, together with a subsequent genetic study (Davison and Field, in prep.), confirm that *L. calceatum* is socially polymorphic within the UK. Nests in the northern UK (Hexham and Inverness) were solitary, while those in the south at Sussex were social (Table 1; Fig. 1a). Sociality at Sussex was characterised by on average 2–3.5 workers per nest and a mean caste-size dimorphism of 6.6 %. The number of workers produced and rate of nest failure differed significantly between years, highlighting the effect of inter-year environmental fluctuations on soil-nesting Hymenoptera. We now discuss social polymorphism and sociality in *L. calceatum*.

### Social phenotype in the UK

Most nests at Sussex (Fig. 1a) were social; at least some B1 females remained at the nest and provisioned a second brood of reproductives. In contrast nests at Hexham and Inverness (Fig. 1a) were solitary; foundresses produced only a single brood of reproductives, which did not become workers and entered directly into hibernation. These results indicate that solitary nests reported by Field (1996) from Dartmoor, an area of southern upland in the UK, are consistent with the altitude-based polymorphism originally reported in Japan by Sakagami and Munakata (1972). Foundresses at Dartmoor and Inverness were smaller than those at Sussex and Hexham, consistent with other sweat bees in which those persisting at higher latitudes or altitudes are smaller (Kirkton 1966; Soucy 2002; Field et al. 2012).

It is likely that sociality in northern and upland areas is precluded because the season is too short for more than one brood (Soucy 2002; Kocher et al. 2014; but see Miyanaga et al. 1999). Indeed, in both 2012 and 2013, foundress-provisioning occurred later at Hexham and Inverness than at Sussex. Similarly, Field et al. (2012) reported that northern *H. rubicundus* foundresses began provisioning considerably later than those in the south, and that their offspring took longer to develop. In both cases B1 offspring in the north probably emerge too late to successfully rear a second brood because nests are initiated later, and cooler temperatures lengthen development time (Table 1; Weissel et al. 2006; Hirata and Higashi 2008). Such constraints probably also limit body size and lead to the significantly smaller size of foundresses from Dartmoor and Inverness relative to those from Sussex (Fig. 5; Field et al. 2012). In light of this it is interesting that foundresses from Hexham and Sussex did not differ in size. One possible explanation is that adult body size follows a saw-tooth cline (Roff 1980; Field et al. 2012). Foundresses in Hexham, possibly just to the north of the transition between social and solitary nesting, might be relatively less time-stressed than those just to the south, because they must rear only a single brood per year instead of two (Field et al. 2012). These foundresses may then be able to capitalise on the relatively longer period of time available for development by providing each offspring with more food, such that development would be lengthened and a larger adult size could be attained. Larger body size is likely to confer benefits such as increased tolerance of cooler temperatures and survival through hibernation (Stone 1994; Brand and Chapuisat 2012, but see Weissel et al. 2012).

At least two nests at Sussex in 2012 were possibly solitary, suggesting that *L. calceatum* can express both social and solitary phenotypes in sympatry as recorded in other polymorphic sweat bees (Packer 1990; Soucy 2002). It seems unlikely that the timing of nest initiation or offspring emergence (e.g. Hirata and Higashi 2008; Field et al. 2010) can explain solitary nesting at Sussex; neither nest was initiated late, and offspring from these nests were among the first and last to emerge (Fig. 3). In some sweat bees, first brood offspring enter hibernation if the foundress has died before they emerge (e.g. Packer 1990; Richards and Packer 1994). However, the foundress was still alive in both solitary nests at Sussex, illustrating that offspring can enter hibernation in the presence of the foundress (see also Hirata and Higashi 2008). Moreover, both foundresses were large, and therefore should not have been at a particular disadvantage in dominance interactions (e.g. Kukuk and May 1991). Yanega (1989, 1993) proposed that mating soon after eclosion could induce offspring to hibernate directly. This hypothesis is impossible to test here, although it has yet to be demonstrated experimentally (Plateaux-Quénu and Packer 1998) and may work only under restrictive conditions (Lucas and Field 2013). Caste-biasing mechanisms are still poorly understood in halictids, and pre-emergence mechanisms mediated via nutrition provided by the foundress cannot be ruled out (Richards and Packer 1994; Brand and Chapuisat 2012).

The B1 female from 2012 seen again in 2013 demonstrates that B1 offspring can successfully overwinter, even though this female did not successfully found a nest in 2013. This observation together with the nests that appeared to become solitary strongly suggests that brood divalency occurs in *L. calceatum*, as is well known in *H*. *rubicundus* (Yanega 1989).

### Foundress-provisioning and B1 offspring

Contrary to the pattern found in *H. rubicundus* by Field et al. (2010), there was no evidence that the B1 offspring of earlier-provisioning foundresses emerged earlier. Instead, the time between a foundresses’ first provision and the emergence of her B1 offspring decreased linearly with date of first provision (Fig. 3). Strikingly, three former co-foundresses in 2013 began provisioning a month after the earliest foundresses, a behaviour known from other sweat bees (Ulrich et al. 2009), but their offspring emerged at a similar time (Fig. 3). Brood develop more rapidly in warmer temperatures (Weissel et al. 2006), and similar patterns in other populations of *H. rubicundus* have been attributed to increased growth rates of later-provisioned offspring, which experience warmer temperatures (Yanega 1993; Field et al. 2012). However, this pattern might also arise if earlier-provisioned offspring do not emerge immediately after eclosion (e.g. Wcislo et al. 1993), or suffered increased mortality relative to later-provisioned brood. Whatever the cause, this relationship suggests that foundresses could not use time of provisioning to anticipate the time of offspring emergence, and hence social phenotype.

Larger foundresses did not produce more workers, or workers that were larger (Fig. 4b), and earlier-provisioning foundresses also did not produce more workers. Earlier-provisioning foundresses might have a longer period during which to produce offspring, and/or larger foundresses should be able to carry more pollen and provision for longer (Stone 1994; Richards 2004), although Field et al. (2012) found no effect of size on foraging parameters in *H. rubicundus*. Larger foundresses produce larger workers in *H. ligatus* (Boomsma and Eickwort 1993; Richards and Packer 1996). However, earlier-emerging *H. ligatus* foundresses appear not to produce more workers (Richards et al. 2015). When provisioning their first brood foundresses must take other factors into account such as increasing risk of mortality or parasitism through foraging, using up resources and potential future reproductive conflict within the nest (Cant and Field 2001; Strohm and Bordon-Hauser 2003; Zobel and Paxton 2007). Foundresses may therefore derive significant genetic benefits from provisioning sufficient offspring to raise a second brood, while remaining alive both to care directly for their own developing B1 offspring (Knerer 1969; Plateaux-Quénu 2008) and to lay B2 eggs (Field et al. 2010). Consequently, foundresses probably cease B1 provisioning at a point that optimises their fitness given these factors.

One possible benefit of large size not assessed here might be increased reproductive dominance within nests (e.g. Breed and Gamboa 1977). Larger foundresses did not produce larger workers (Fig. 4b), and therefore caste-size dimorphism was greatest in nests with the largest foundresses. In nests of *H. ligatus*, foundresses that were the largest relative to their workers appeared to gain a greater share of reproduction (Richards et al. 1995; Richards and Packer 1996). This may not be the case in all species, however, as reproduction was successfully monopolised in nests of *H*. *rubicundus* where workers and egg-layers did not differ in size (Field et al. 2010). The future application of genetic markers to B2 offspring will help to resolve this issue in *L. calceatum* (Davison and Field in prep.).

Results from this study provide further evidence that caste-size dimorphism in *L. calceatum* is geographically variable. In France foundresses are on average 8.3–13 % larger than workers (Plateaux-Quénu 1992), whereas in Japan, Sakagami and Munakata (1972) reported 3.5–5.5 %, and in the present study mean caste-size dimorphism was 6.6 %. The reason for such differences remains unclear, but it could be that caste-size dimorphism is greater in areas where bees experience warmer temperatures (Sakagami and Munakata 1972; Soucy 2002), a pattern generated experimentally by Plateaux-Quénu and Plateaux (1980). This could be because the longer growing season further south allows the production of larger reproductives. If there is little advantage in also producing larger workers (Strohm and Liebig 2008), worker size may remain constant or at least increase at a slower rate. Therefore, caste-size dimorphism would be larger at lower latitudes and could explain the discrepancy in measurements reported between the present study and France.

### Environmental effects on nesting success

Inter-year variation in environmental conditions can significantly affect brood survival and demography (Richards and Packer 1996). In the present study, the rate of nest failure was significantly greater, and the mean number of workers produced significantly fewer, in 2012 than 2013. Co-founded nests were significantly less likely to fail, as previously found in sweat bees and polistine wasps (Richards and Packer 1998; Tibbetts and Reeve 2003). Development of B1 offspring occurred mostly during June and early July (Fig. 2), which in 2012 were considerably wetter than 2013 (Fig. 1b). In 2012, 84 % of nests failed to produce any detected B1 offspring, whereas in 2013 this was only 38 %. It is possible that the higher rate of nest failure and smaller brood sizes in 2012 resulted from increased brood mortality due to the intense summer rainfall. Poor weather is unlikely to have caused foundresses to provision fewer B1 offspring in 2012, because most foundresses provisioned during the warm and sunny weeks of May. Moreover, workers were the same size on both years, suggesting that similar resources were available to foundresses during provisioning in 2012 and 2013 (Richards 2004; Richards et al. 2015).

High rates of nest failure are common in halictids (Ulrich et al. 2009), particularly during the foundress phase (Sakagami and Fukuda 1989) and can be accentuated by cool and wet weather causing brood to become mouldy (Richards and Packer 1995; Soucy 2002). Nevertheless, brood cells in *L. calceatum* are clustered and surrounded by a cavity (Packer and Knerer 1985). The cavity is thought to mitigate the effects of rainfall by improving drainage (Packer and Knerer 1985; Packer 1990), and therefore it is perhaps surprising that the high rainfall in 2012 resulted in such a considerable rate of nest failure in 2012. Our results highlight how strongly weather conditions can influence reproductive success of ground-nesting Hymenoptera.

### Social level in *L. calceatum*

Across both years at Sussex, mean within-nest caste-size dimorphism was 6.6 %, and foundresses produced a mean of two and 3.5 workers in 2012 and 2013, respectively. Both figures are small in comparison with more specialised obligately social species in the *Lasioglossum* subgenus *Evylaeus* (Packer and Knerer 1985; Wyman and Richards 2003; Fig. 2.7). Although foundresses were significantly larger than workers there was still considerable overlap in size. Obligately eusocial species such as *L. malachurum* not only produce more workers but also show distinct and almost non-overlapping bimodality in caste sizes (Knerer 1980; Wyman and Richards 2003), reflecting an increased specialisation for social nesting. The significant cross-species correlation between group size and caste-size dimorphism may reflect an adaptation to reduce kin conflict if foundresses more easily behaviourally dominate a larger number of smaller workers (Kukuk and May 1991; Bourke and Franks 1995).

The ability to nest solitarily is not necessarily lost in obligate eusocial nesters (e.g. see Rehan et al. 2013), but polymorphism probably limits the degree to which social behaviour can become specialised. Nevertheless, *L. calceatum* may be more socially specialised than other polymorphic species. For example, *L. calceatum* nests socially in areas where its polymorphic sister species *L. albipes* is solitary (Plateaux-Quénu et al. 2000), and B1 females at Sussex became workers later in the season than B1 offspring of *H*. *rubicundus* (Field et al. 2010). Together, this suggests that sociality in *L. calceatum* may occur over a wider range of conditions than in other polymorphic species; perhaps suggesting that social phenotype may be less plastic. Field transplantation experiments (Field et al. 2010, 2012) will be required to ascertain the existence or extent of social plasticity in *L. calceatum*. Interestingly, *L. albipes* is thought not to be plastic (Plateaux-Quénu et al. 2000).

## Electronic supplementary material

Below is the link to the electronic supplementary material.

Supplementary material 1 (PDF 301 kb)

## References

[CR1] Bates D, Maechler M, Bolker B and Walker S (2015) lme4: Linear mixed-effects models using Eigen and S4. R package version 1.1–8, http://CRAN.R-project.org/package=lme4. Accessed 20 Feb 2015

[CR2] Boesi CP (2009). Searching for the right target: oviposition and feeding behavior in *Bombylius* bee flies (Diptera: Bombyliidae). Zool Stud.

[CR3] Bogusch P, Kratochvíl L, Straka J (2006). Generalist cuckoo bees (Hymenoptera: Apoidea: *Sphecodes*) are species-specialist at the individual level. Behav Ecol Sociobiol.

[CR4] Bonelli B (1965). Osservazioni sugli imenotteri melliferi e predatori della Val di Fiemme VI. *Halictus calceatus* Scop. (sin. *Lasioglossum cylindricum* F.). Studi Trentini Sci Nat.

[CR5] Bonelli B (1968). Osservazioni biologiche sugli imenotteri melliferi e predatori Val di Fiemme XXVIII contributo. *Halictus calceatus* Scopoli (Hymenoptera: Halictidae). Studi Trentini Sci Nat.

[CR6] Boomsma JJ, Eickwort G (1993). Colony structure, provisioning and sex allocation in the sweat bee *Halictus ligatus* (Hymenoptera: Halictidae). Biol J Linn Soc.

[CR7] Bourke AFG (1999). Colony size, social complexity and reproductive conflict in social insects. J Evol Biol.

[CR8] Bourke AFG, Franks NR (1995). Social evolution in ants.

[CR9] Brand N, Chapuisat M (2012). Born to be bee, fed to be worker? The caste system of a primitively eusocial insect. Front Zool.

[CR10] Brand N, Chapuisat M (2016). Low relatedness and frequent inter-nest movements in a eusocial sweat bee. Insect Soc.

[CR11] Breed MD, Gamboa GJ (1977). Control of worker activities by queen behaviour in a primitively eusocial bee. Science.

[CR12] Cant MA, Field J (2001). Helping effort and future fitness in cooperative animal societies. Proc R Soc B.

[CR13] Chapuisat M (2010). Evolution: plastic sociality in a sweat bee. Curr Biol.

[CR16] Eickwort GC, Eickwort JM, Gordon J, Eickwort MA, Wcislo WT (1996). Solitary behavior in a high-altitude population of the social sweat bee *Halictus rubicundus* (Hymenoptera: Halictidae). Behav Ecol Sociobiol.

[CR17] Field J (1996). Patterns of provisioning and iteroparity in a solitary halictine bee *Lasioglossum* (*Evylaeus*) *fratellum* (Perez), with notes on *L.* (*E.*) *calceatum* (Scop.) *L*. (*E*.) villosulum (K.). Insect Soc.

[CR18] Field J, Paxton RJ, Soro A, Bridge C (2010). Cryptic plasticity underlies a major evolutionary transition. Curr Biol.

[CR19] Field J, Paxton R, Soro A, Craze P, Bridge C (2012). Body size, demography and foraging in a socially plastic sweat bee: a common garden experiment. Behav Ecol Sociobiol.

[CR20] Hay SI, Tatem AJ, Graham AJ, Goetz SJ, Rogers DJ, Hay SI, Graham AJ, Rogers DJ (2006). Global environmental data for mapping infectious disease distribution. Advances in parsitology.

[CR21] Hirata M, Higashi S (2008). Degree-day accumulation controlling allopatric and sympatric variations in the sociality of sweat bees, *Lasioglossum (Evylaeus) baleicum* (Hymenoptera: Halictidae). Behav Ecol Sociobiol.

[CR22] Kirkton RM (1966) Biosystematic analysis of variation of *Halictus* (*Halictus*) *ligatus* Say (Hymenoptera: Halictidae). Ph.D. Thesis, Purdue University

[CR23] Knerer G (1969). Brood care in halictine bees. Science.

[CR24] Knerer G (1980). Evolution of halictine castes. Naturwissenschaften.

[CR25] Kocher SD, Pellissier L, Veller C, Purcell J, Nowak MA, Chapuisat M, Pierce NE (2014). Transitions in social complexity along elevational gradients reveal a combined impact of season length and development time on social evolution. Proc R Soc B.

[CR26] Kukuk P, May B (1991). Colony dynamics in a primitively eusocial halictine bee *Lasioglossum* (*Dialictus*) *zephyrum* (Hymenoptera: Halictidae). Insect Soc.

[CR27] Lucas ER, Field J (2013). Caste determination through mating in primitively eusocial societies. J Theor Biol.

[CR28] Maynard Smith J, Szathmáry E (1995). The major transitions in evolution.

[CR29] Miyanaga R, Maeta Y, Sakagami SF (1999). Geographical variation of sociality and size-linked color patterns in *Lasioglossum (Evylaeus) apristum* (Vachal) in Japan (Hymenoptera: Halictidae). Insect Soc.

[CR30] Packer L (1990). Solitary and eusocial nests in a population of *Augochlorella striata* (Provancher) (Hymenoptera: Halictidae) at the northern edge of its range. Behav Ecol Sociobiol.

[CR31] Packer L, Knerer G (1985). Social evolution and its correlates in bees of the subgenus *Evylaeus*. Behav Ecol Sociobiol.

[CR101] Pesenko YA, Banaszak J, Radchenko VG, Cierzniak, T (2000) Bees of the family Halictidae (excluding Sphecodes) of Poland: taxonomy, ecology, bionomics. Wydawnictwo Uczelniane Wyższej Szkoły Pedagogicznej w Bydgoszczy, Bydgoszcz

[CR32] Plateaux-Quénu C (1992). Comparative biological data in two closely related eusocial species *Evylaeus calceatus* (Scop.) and *Evylaeus albipes* (F.) (Hym., Halictinae). Insect Soc.

[CR33] Plateaux-Quénu C (2008). Subsociality in halictine bees. Insect Soc.

[CR34] Plateaux-Quénu C, Packer L (1998). A test of the mating limitation hypothesis for caste determination in Evylaeus albipes (Hymenoptera: Halictidae), a primitively eusocial halictine bee. J Insect Behav.

[CR35] Plateaux-Quénu C, Plateaux L (1980). Action de la température sur la taille, le sexe et le cycle des individus depremiere eouvée chez *Evylaeus calceatus* (Scop.) (Hym., Halictinae): premiere étude expérimentale. Ann Des Sci Nat Compr Zool.

[CR36] Plateaux-Quénu C, Plateaux L, Packer L (2000). Population-typical behaviours are retained when eusocial and non-eusocial forms of *Evylaeus albipes* (F.) (Hymenoptera: Halictidae) are reared simultaneously in the laboratory. Insect Soc.

[CR53] R Core Team (2013) R: a language and environment for statistical computing. R Foundation for Statistical Computing, Vienna, Austria. URL http://www.R-project.org/. Accessed 20 Oct 2013

[CR37] Rehan SM, Rotella A, Onuferko TM, Richards MH (2013). Colony disturbance and solitary nest initiation by workers in the obligately eusocial sweat bee, *Halictus ligatus*. Insect Soc.

[CR38] Richards MH (2004). Annual and social variation in foraging effort of the obligately eusocial sweat bee, *Halictus ligatus* (Hymenoptera: Halictidae). J Kansas Entom.

[CR39] Richards MH, Packer L (1994). Trophic aspects of caste determination in *Halictus ligatus*, a primitively eusocial sweat bee. Behav Ecol Sociobiol.

[CR40] Richards MH, Packer L (1995). Annual variation in survival and reproduction of the primitively eusocial sweat bee *Halictus ligatus* (Hymenoptera: Halictidae). Can J Zool.

[CR41] Richards MH, Packer L (1996). The socioecology of body size variation in the primitively eusocial sweat bee, *Halictus ligatus* (Hymenoptera: Halictidae). Oikos.

[CR42] Richards MH, Packer L (1998). Demography and relatedness in multiple-foundress nests of the social sweat bee, *Halictus ligatus*. Insect Soc.

[CR43] Richards MH, Packer L, Seger J (1995). Unexpected patterns of parentage and relatedness in a primitively eusocial bee. Nature.

[CR44] Richards MH, Onuferko TM, Rehan SM (2015). Phenological, but not social, variation associated with climate differences in a eusocial sweat bee, *Halictus ligatus*, nesting in southern Ontario. J Hymenopt Res.

[CR45] Roff D (1980). Optimizing development time in a seasonal environment—the ups and downs of clinal variation. Oecologia.

[CR46] Sakagami S, Fukuda H (1989). Nest founding and nest survival in a eusocial halictine bee, *Lasioglossum duplex.* Additional observations. Res Popul Ecol.

[CR47] Sakagami SF, Munakata M (1972). Distribution and bionomics of a transpalearctic eusocial halictine bee, *Lasioglossum* (*Evylaeus*) *calceatum*, in Northern Japan, with reference to its solitary life cycle at high altitude. Journal of the Faculty of Science, Hokkaido University, Series 6. Zoology.

[CR48] Schwarz MP, Richards MH, Danforth BN (2007). Changing paradigms in insect social evolution: insights from halictine and allodapine bees. Ann Rev Entomol.

[CR49] Soucy SL (2002). Nesting biology and socially polymorphic behavior of the sweat bee Halictus rubicundus (Hymenoptera: Halictidae). Ann Entomol Soc Am.

[CR100] Soucy SL, Danforth BN (2002). Phylogeography of the socially polymorphic sweat bee *Halictus rubicundus* (Hymenoptera: Halictidae). Evolution.

[CR50] Stone GN (1994). Activity patterns of females of the solitary bee *Anthophora plumipes* in relation to temperature, nectar supplies and body size. Ecol Entomol.

[CR51] Strohm E, Bordon-Hauser A (2003). Advantages and disadvantages of large colony size in a halictid bee: the queen’s perspective. Behav Ecol.

[CR52] Strohm E, Liebig J, Korb JH, Heinze J (2008). Why are so many bees but so few digger wasps social? The effect of provisioning mode and worker efficiency on the distribution of sociality among the Apoidea. Ecology of social evolution.

[CR54] Tibbetts EA, Reeve HK (2003). Benefits of foundress associations in the paper wasp *Polistes dominulus*: increased productivity and survival, but no assurance of fitness returns. Behav Ecol.

[CR55] Ulrich Y, Perrin N, Chapuisat M (2009). Flexible social organization and high incidence of drifting in the sweat bee, *Halictus scabiosae*. Mol Ecol.

[CR56] Wcislo WT, Choe JC, Crespi B (1997). Behavioral environments of the sweat bees. Social behaviour in insects and arachnids.

[CR57] Wcislo WT, Wille A, Orozco E (1993). Nesting biology of tropical solitary and social sweat bees, *Lasioglossum* (*Dialictus*) *figueresi* Wcislo and *L.* (*D.*) *aeneiventre* (Friese) (Hymenoptera: Halictidae. Insect Soc.

[CR58] Weissel N, Mitesser O, Liebig J, Poethke HJ, Strohm E (2006). The influence of soil temperature on the nesting cycle of the halictid bee *Lasioglossum malachurum*. Insect Soc.

[CR59] Weissel N, Mitesser O, Poethke HJ, Strohm E (2012). Availability and depletion of fat reserves in halictid foundress queens with a focus on solitary nest founding. Insect Soc.

[CR60] Wyman LM, Richards MH (2003). Colony social organization of *Lasioglossum malachurum* Kirby (Hymenoptera: Halictidae) in southern Greece. Insect Soc.

[CR61] Yagi N, Hasegawa E (2012). A halictid bee with sympatric solitary and eusocial nests offers evidence for Hamilton’s rule. Nat Comun.

[CR62] Yanega D (1988). Social plasticity and early diapausing females in a primitively social bee. PNAS.

[CR63] Yanega D (1989). Caste determination and differential diapause within the first brood of *Halictus rubicundus* in New York (Hymenoptera: Halictidae). Behav Ecol Sociobiol.

[CR64] Yanega D (1993). Environmental influences on male production and social structure in *Halictus rubicundus* (Hymenoptera: Halictidae). Insect Soc.

[CR65] Zobel MU, Paxton RJ (2007). Is big the best? Queen size, usurpation and nest closure in a primitively eusocial sweat bee (*Lasioglossum malachurum*). Behav Ecol Sociobiol.

