# Supplementary material for: Social polymorphism in the sweat bee Lasioglossum (Evylaeus) calceatum
Source: Insectes Soc. 2016 May 10;63:327–38. doi: 10.1007/s00040-016-0473-3 (PMC4869766; doi:10.1007/s00040-016-0473-3)
Supplement: Supplementary file 1 — Supplementary material 1 (PDF 301 kb) [file 40_2016_473_MOESM1_ESM.pdf]

Supplementary material

P. J. Davison and J. Field

Social polymorphism in the sweat bee *Lasioglossum* (*Evylaeus*) *calceatum* (Hymenoptera: Halictidae)

Insectes Sociaux

School of Life Sciences, University of Sussex, John Maynard Smith Building, Brighton, BN1 9QG, UK

Corresponding author: Paul James Davison

Email: p.j.davison@sussex.ac.uk

Phone: 01273 872764

Table S1
Results from Tukey's HSD individual pairwise comparisons of foundress size from different populations

Comparison	p-value
Hexham-Dartmoor	0.002
Inverness-Dartmoor	0.994
Sussex-Dartmoor	0.001
Inverness-Hexham	0.002
Sussex-Hexham	0.818
Sussex-Inverness	0.002

Table S2
Details of species, mean number of workers, caste-size dimorphism and method of measurements used for data included in the *Evylaeus* social level analysis

Species	Mean number of workers	Caste-size dimorphism (%)	Environment studied	Measurement	Method of counting worker number	Reference
Lasioglossum laticeps	4.1	7.3	wild	wing	census	Packer and Knerer, 1985
L. pauxillum	4	14.5	wild	wing	census	Packer and Knerer, 1985
L. lineare	4.1	15	wild	wing	census	Packer and Knerer, 1985
L. lineare	6.3	21	wild	wing	census	Packer and Knerer, 1985
L. malachurum	6.7	14.3	wild	wing	census	Packer and Knerer, 1985
L. malachurum	6.8	18	wild	wing	census	Packer and Knerer, 1985
L. malachurum	5.1	17.5	wild	wing	census	Packer and Knerer, 1985
L. malachurum	6.5	18.1	wild	wing	census	Packer and Knerer, 1985
L. malachurum	4.5	15	wild	wing	census	Unpublished
L. malachurum	4	14.3	wild	wing	census	Richards, 2000
L. malachurum	7	10.3	wild	head	census	Wyman & Richards, 2003
L. nigripes	7.2	10.3	wild	head	census	P&K 1985
L. marginatum	3.5	0.1	wild	wing	census	Packer and Knerer, 1985
L. calceatum	2	6.9	wild	wing	census	Present study
L. calceatum	3.5	6.5	wild	wing	census	Present study
L. duplex	4.6	9.6	wild	head	census	Packer and Knerer, 1985
L. duplex	1.3	8	wild	head	census	Hirata et al. 2005
L. duplex	4.1	4.5	wild	head	census	Hirata et al. 2005
L. duplex	3.6	6.12	wild	head	census	Hirata et al. 2005
L. baleicum	1.7	4.5	wild	head	census	Cronin & Hirata, 2003
L. baleicum	1.17	0	wild	head	census	Yagi & Hasegawa, 2012
L. baleicum	4.4	9.3	wild	head	census	Hirata and Higashi, 2008
L. albipes	3	5.6	lab	head	census	Plateaux-Quénu, 1992
L apristum	6.9	7.7	greenhouse	head	census	Miyanaga et al. 1999
